# Germline copy number variants and endometrial cancer risk

**DOI:** 10.1007/s00439-024-02707-9

**Published:** 2024-11-04

**Authors:** Cassie E. Stylianou, George A. R. Wiggins, Vanessa L. Lau, Joe Dennis, Andrew N. Shelling, Michelle Wilson, Peter Sykes, Frederic Amant, Daniela Annibali, Wout De Wispelaere, Douglas F. Easton, Peter A. Fasching, Dylan M. Glubb, Ellen L. Goode, Diether Lambrechts, Paul D. P. Pharoah, Rodney J. Scott, Emma Tham, Ian Tomlinson, Manjeet K. Bolla, Fergus J. Couch, Kamila Czene, Thilo Dörk, Alison M. Dunning, Olivia Fletcher, Montserrat García-Closas, Reiner Hoppe, ABCTB Investigators, Christine Clarke, Deborah Marsh, Rodney Scott, Robert Baxter, Desmond Yip, Jane Carpenter, Alison Davis, Nirmala Pathmanathan, Peter Simpson, J Dinny Graham, Mythily Sachchithananthan, Helena Jernström, Rudolf Kaaks, Kyriaki Michailidou, Nadia Obi, Melissa C. Southey, Jennifer Stone, Qin Wang, Amanda B. Spurdle, Tracy A. O’Mara, John Pearson, Logan C. Walker

**Affiliations:** 1 Department of Pathology and Biomedical Science, University of Otago, Christchurch, New Zealand; 2 Department of Public Health and Primary Care, Centre for Cancer Genetic Epidemiology, University of Cambridge, Cambridge, UK; 3 Department of Obstetrics and Gynaecology, University of Auckland, Auckland, New Zealand; 4 Te Pūriri o Te Ora Regional Cancer and Blood Service, Auckland Hospital, Auckland, New Zealand; 5 Department of Obstetrics and Gynaecology, University of Otago, Christchurch, New Zealand; 6 Division of Gynecologic Oncology, Department of Obstetrics and Gynecology, University Hospitals KU Leuven, University of Leuven, Leuven, Belgium; 7 Gynecological Oncology Laboratory, Department of Oncology, KU Leuven and Leuven Cancer Institute (LKI), Leuven, Belgium; 8 Department of Oncology, Centre for Cancer Genetic Epidemiology, University of Cambridge, Cambridge, UK; 9 Department of Gynecology and Obstetrics, Comprehensive Cancer Center Erlangen-EMN, University Hospital Erlangen, Friedrich-Alexander University Erlangen-Nuremberg, Erlangen, Germany; 10 Cancer Research Program, QIMR Berghofer Medical Research Institute, Brisbane, QLD Australia; 11 Division of Epidemiology, Department of Quantitative Health Sciences, Mayo Clinic, Rochester, MN USA; 12 Laboratory for Translational Genetics, Department of Human Genetics, KU Leuven, Leuven, Belgium; 13 VIB Center for Cancer Biology, VIB, Leuven, Belgium; 14 Department of Computational Biomedicine, Cedars-Sinai Medical Center, West Hollywood, CA USA; 15 Division of Molecular Medicine, Pathology North, John Hunter Hospital, Newcastle, NSW Australia; 16 Faculty of Health, Discipline of Medical Genetics, School of Biomedical Sciences and Pharmacy, University of Newcastle, Callaghan, NSW Australia; 17 Hunter Medical Research Institute, John Hunter Hospital, Newcastle, NSW Australia; 18 Department of Molecular Medicine and Surgery, Karolinska Institutet, Stockholm, Sweden; 19 Clinical Genetics and Genomics, Karolinska University Hospital, Stockholm, Sweden; 20 Department of Oncology, University of Oxford, Oxford, UK; 21 Department of Laboratory Medicine and Pathology, Mayo Clinic, Rochester, MN USA; 22 Department of Medical Epidemiology and Biostatistics, Karolinska Institutet, Stockholm, Sweden; 23 Gynaecology Research Unit, Hannover Medical School, Hannover, Germany; 24 The Breast Cancer Now Toby Robins Research Centre, The Institute of Cancer Research, London, UK; 25 Division of Genetics and Epidemiology, The Institute of Cancer Research, London, UK; 26 Dr. Margarete Fischer-Bosch-Institute of Clinical Pharmacology, Stuttgart, Germany; 27 University of Tübingen, Tübingen, Germany; 28 Australian Breast Cancer Tissue Bank, Westmead Institute for Medical Research, University of Sydney, Sydney, NSW Australia; 29 Oncology, Department of Clinical Sciences in Lund, Lund University, Lund, Sweden; 30 Division of Cancer Epidemiology, German Cancer Research Center (DKFZ), Heidelberg, Germany; 31 Biostatistics Unit, The Cyprus Institute of Neurology and Genetics, Nicosia, Cyprus; 32 Institute for Occupational and Maritime Medicine, University Medical Center Hamburg-Eppendorf, Hamburg, Germany; 33 Institute for Medical Biometry and Epidemiology, University Medical Center Hamburg-Eppendorf, Hamburg, Germany; 34 Precision Medicine, School of Clinical Sciences at Monash Health, Monash University, Clayton, VIC Australia; 35 Department of Clinical Pathology, The University of Melbourne, Melbourne, VIC Australia; 36 Cancer Epidemiology Division, Cancer Council Victoria, Melbourne, VIC Australia; 37 Genetic Epidemiology Group, School of Population and Global Health, University of Western Australia, Perth, WA Australia; 38 Centre for Epidemiology and Biostatistics, Melbourne School of Population and Global Health, The University of Melbourne, Melbourne, VIC Australia; 39 Public Health Program, QIMR Berghofer Medical Research Institute, Brisbane, QLD Australia; 40 Department of Medicine, University of Otago, Christchurch, New Zealand

## Abstract

Known risk loci for endometrial cancer explain approximately one third of familial endometrial cancer. However, the association of germline copy number variants (CNVs) with endometrial cancer risk remains relatively unknown. We conducted a genome-wide analysis of rare CNVs overlapping gene regions in 4115 endometrial cancer cases and 17,818 controls to identify functionally relevant variants associated with disease. We identified a 1.22-fold greater number of CNVs in DNA samples from cases compared to DNA samples from controls (*p* = 4.4 × 10^–63^). Under three models of putative CNV impact (deletion, duplication, and loss of function), genome-wide association studies identified 141 candidate gene loci associated (*p* < 0.01) with endometrial cancer risk. Pathway analysis of the candidate loci revealed an enrichment of genes involved in the 16p11.2 proximal deletion syndrome, driven by a large recurrent deletion (chr16:29,595,483-30,159,693) identified in 0.15% of endometrial cancer cases and 0.02% of control participants. Together, these data provide evidence that rare copy number variants have a role in endometrial cancer susceptibility and that the proximal 16p11.2 BP4-BP5 region contains 25 candidate risk gene(s) that warrant further analysis to better understand their role in human disease.

**Supplementary Information:**

The online version contains supplementary material available at 10.1007/s00439-024-02707-9.

## Introduction

Endometrial cancer is it the most commonly diagnosed gynaecological cancer in developed countries (Rodríguez-Palacios et al. 2022). The incidence of endometrial cancer has been increasing, and a key contributor to this trend is the rising prevalence of obesity, a major risk factor for this disease. Other risk factors include reproductive risk factors such as early menarche, late menopause and nulliparity, exogenous oestrogen use, and a family history of endometrial or colorectal cancer (Lortet-Tieulent et al. 2018). While much progress has been made to understand the biology of endometrial cancer, the genetic risk factors underlying this disease have not been fully elucidated.

Genetic risk factors for endometrial cancer include inherited pathogenic variants DNA mismatch repair (MMR) genes associated with Lynch Syndrome (*MLH1, MSH2*, *MSH6* and *PMS2*) and the tumour suppressor *PTEN.* Genome-wide technologies, such as single nucleotide polymorphisms (SNP)-arrays have identified common risk loci associated with endometrial cancer that confer levels of risk (odds ratio [OR] < 2), and in aggregate explain less than a third of the estimated familial relative risk for endometrial cancer (Chen et al. 2016; O’Mara et al. 2018; Wang et al. 2022).

Copy number variants (CNVs) are a form of structural variation that are pervasive in the human genome and can disrupt gene function by altering gene dosage, coding sequence or regulation. The de novo mutation rate of CNVs is several orders of magnitude higher than the mutation rate of single nucleotide variants (Zhang et al. 2009). However, CNVs are typically rare which is consistent with the hypothesis that CNVs can be pathogenic and therefore often under negative selection. A recent study of CNVs in 100,000 individuals with European ancestry showed that > 98.5% CNV variants had a minor allele frequency < 0.01 (Li et al. 2020).

Rare pathogenic CNVs have previously been identified in cancer susceptibility genes, including known endometrial cancer syndrome genes (Truty et al. 2019). In a *MSH2-*associated Lynch syndrome cohort (n = 83), 11% of pathogenic variants identified in *MSH2* were CNVs (Romero et al. 2013). Similarly, single to multi-exon deletions make up 22% of pathogenic variants in *PMS2*, 21% of pathogenic variants in *MSH2* and *MLH1* and 4% of pathogenic variants in *MSH6* (Lagerstedt-Robinson et al. 2016). In a genome-wide analysis of 1209 endometrial cancer cases and 528 cancer-unaffected female controls, we previously reported that rare deletions of likely functional genomic regions (e.g. exons and CpG islands) were more frequent in cases compared to controls (Moir-Meyer et al. 2015). These results implicated rare germline deletions of functional and regulatory genomic regions as mechanisms for conferring risk of endometrial cancer.

To identify endometrial cancer CNV risk loci, we performed a gene-centric genome-wide association study (GWAS) using the OncoArray single nucleotide polymorphism (SNP) array on a large cohort (n = 21,933) of endometrial cancer cases and healthy controls with European ancestry. Additionally, we conducted analysis of global CNV burden in endometrial cancer cases compared to controls.

## Methods

### Study cohort and genotyping

The study cohort was comprised of female individuals from 28 studies, with cases sourced via the Endometrial Cancer Association Consortium (ECAC) and healthy female controls from the Breast Cancer Association Consortium (Supplementary Table S1). The characteristics of the cohorts have been previously described (O’Mara et al. 2018). DNA samples derived from whole blood were genotyped on the Infinium OncoArray-500K Beadchip (Illumina) across five genotyping facilities, all participants were of European descent. The OncoArray consists of 533,631 probes, half of which were selected from the HumanCore (Illumina) backbone and the other half placed in regions previously associated with cancer risk (Amos et al. 2017).

### CNV calling

CNVs were called using CamCNV, a method designed to confidently call rare (MAF < 3%) CNVs with fewer probes and higher confidence (Dennis et al. 2021). Quality control was performed for samples and CNVs (Supplementary Table S2). Briefly, for each sample a derivative log ratio spread (DLRS) figure was calculated as the average variance in Log R Ratio (LRR) intensities of neighbouring probes by genome position over the whole genomes (Cooper et al. 2015). Samples with a DLRS Fig. 3.5 SD above the DLRS study mean (DLRS = 0.2) were removed. Principle component adjustment (PCA) of the LRR intensities at each probe was then performed to reduce batch effects in probe intensity and adjust for variation in hybridisation intensity (genomic waves) (Diskin et al. 2008). Following PCA, a second DLRS sample exclusion was applied, again removing samples with a DLRS 3.5 SD above post PCA-adjusted sample mean of DLRS = 0.1. Samples with excessive heterogeneity (4.89 SD from the study mean), or those with sex chromosome abnormalities were also excluded from study (Michailidou et al. 2017). Prior to CNV calling, probes with data that failed to be clustered by Illumina Gentrain algorithm (< 0.15), low intensity probes (mean intensity < 0.2) or any with high LRR variance (two SD above the mean variance of all probes) were removed. Additionally, CNVs predicted within immune-related loci (Immunoglobin heavy chain, T-cell receptor and major histocompatibility complex) or near centromeres and telomeres were also excluded. Only CNVs called using 3–200 probes were retained. Previous published thresholds of excess germline CNV count in human blood ranged between 30 and 200 CNVs (Aguirre et al. 2019; Macé et al. 2016). We adopted a lower threshold and excluded samples predicted to carry n ≥ 50 (Supplementary Table S2). The final analysis dataset included data for 4,115 endometrial cancer cases (371 removed) and 17,818 controls (1,073 removed).

### CNV annotation

CNVs were annotated for overlap with protein coding genes and exons sourced using biomaRt and EnsDB (Hsapiens.v75) R packages, with the largest Ensembl transcript used to define gene boundaries (Durinck et al. 2009; Rainer 2017). All genomic features were restricted to chromosomes 1–23/X, and any elements mapping to alternative chromosomes (i.e., sequence scaffolds or mitochondrial chromosomes) were excluded from analysis. Genomic coordinates were based on the GRCh37/hg19 genome build. In situations where genomic data was in an alternative genome build, the UCSC LiftOver tool was used for conversion to GRCH37/hg19 (https://genome.ucsc.edu/cgi-bin/hgLiftOver). All CNVs were assessed for overlap (≥ 1 bp) with regions of interest in R using the GenomicRanges package (V1.4) (Lawrence et al. 2013).

### CNV burden

CNV burden was estimated between endometrial cancer cases and controls for: total number of CNVs, the number of genic CNVs, the number of exonic CNVs and number of intergenic CNVs, respectively. Each burden analysis was repeated for CNV deletions, CNV duplications and all CNVs. Statistical significance of differences in CNV burden between cases and controls were determined by a two-sided Student’s *t*-test, *p*-values < 0.05 were considered statistically significant.

### Copy number variation (CNV)-GWAS

Associations between CNVs and endometrial cancer were assessed by performing a gene-specific test using gene boundaries to define regions of interest. Case and control CNV overlap frequency was determined for each gene region and association was tested by fitting a binomial logistic regression model. Given the varying modes of effects from copy number gain and copy number loss, deletions and duplications were tested independently. Additionally, models were estimated on putative loss of function. A CNV was included in the loss of function GWAS if it was either predicted as a deletion or a duplication that partially overlapped a gene region. A genome-wide significance threshold was calculated for each GWAS conducted: this was represented as 0.05/6014, 0.05/8377 and 0.05/8613 for deletion-only, duplication-only and loss of function respectively.

Additionally, to explicitly model the level of evidence for genes already associated with endometrial cancer, the Bayesian false discovery probability (BFDP) approach was applied (Wakefield 2008) with the prior probabilities assigned at 0.5, for the genes associated with Lynch syndrome, 0.2 for genes with previous associations and 0.05 for genes with little to no prior evidence (Supplementary Tables S5-S7). An upper bound of 8.0 was applied on the odds ratio for any association, all parameters were chosen to reflect the rare nature and large effect of the tested CNV. Lastly, associations at *p* < 0.01 were considered as candidate associations.

### Overlap with previously identified risk SNPs

SNPs associated with disease risk were directly downloaded from the NHGRI-EBI GWAS Catalog (accessed Jan 2024) for the following traits; endometrial cancer (MONDO_0011962, n = 84), Type 2 Diabetes (MONDO_0005148, n = 3516) and Obesity (EFO_0001073, n = 297). SNP associated with these traits were expanded to include any variant in linkage disequilibrium (LD, R^2^ > 0.8) in the ‘EUR’ population from 1000 genomes. Germline CNVs overlapping candidate endometrial cancer risk genes were first assessed for direct overlap with SNP, and the candidate gene list was compared to GWAS mapped gene(s).

### Pathway analysis

Over-representation analysis was performed in R v3.14 using the gProfiler2 package by applying a hypergeometric test to assess enrichment, all results presented are Bonferroni corrected (Kolberg et al. 2020). To allow for variation among candidate endometrial cancer risk genes (*p* < 0.01) derived from different GWAS, top hits from each GWAS were assessed independently. Additionally, FUMA-GWAS was used to test if candidate genes were enriched for genes reported in the GWAS (Watanabe et al. 2017).

### Expression in endometrial tissue and dosage sensitivity

Expression of candidate genes was assessed in normal and tumour tissue using publicly available data. The R packages hpar and ExperimentHub were used to retrieve RNA levels (Transcripts per million (TPM)) directly from the Human Protein Atlas repository (L and Martin 2022; Morgan and Shepherd 2022). Genes were grouped into expression categories using thresholds defined by Expression Atlas (Papatheodorou et al. 2018). Dosage sensitivities of candidate genes were assessed using mRNA expression data and putative copy number of genes from The Cancer Genome Atlas- Uterine Corpus Endometrial Carcinoma (TCGA-UCEC) dataset using the cBioPortalData package from R (Bonneville et al. 2017; Ramos et al. 2020). Candidate risk genes were deemed dosage sensitive if there was a positive, significant (P < 0.0001) relationship between copy number and expression.

### CNV validation

Accessible whole-blood DNA samples from the study cohort were used to validate 17 putative CNV regions. CNV validation was carried out using NanoString nCounter (NanoString Technologies, Inc) following the manufacturer’s protocol. Custom Nanostring probes for CNV regions are listed in Supplementary Table S3. Where possible, three independent probe pairs were designed for each CNV unless the region was too small to accommodate, in which case two probes were used. nSolver 4.0 analysis software was used to perform quality control on raw counts and normalised to a set of invariant control probe pairs. CNVs were partitioned by carrier status and count ratios were calculated to call CNV status.

## Results

### Identification of CNVs in the study cohort

A total of 63,349 rare deletions and 48,555 rare duplications were identified across the 21,933 study participants, of which 46,234 were unique (25,047 deletions and 21,187 duplications). On average, duplications were 2.4 times larger than deletions (mean length 99 kilobases (kb) for duplications vs 41 kb for deletions). In total, 10,637 unique protein coding genes were predicted to be encompased by 24,390 unique CNVs, with 40.7% of deletions and 52.7% of duplications predicted to overlap at least one gene region (Supplementary Table S4). On average, we identified 5.10 CNVs per sample (range = 0–47) and 2.34 genic CNVs per sample (range = 0–47), with 96.3% of samples estimated to carry at least one CNV. The highest minor allele frequency for CNVs called with CamCNV was 2.2%. The majority of CNVs (79% of deletions and 81% of duplications) identified were only identified in a single sample (allele frequency = 0.0045%) highlighting the uniqueness of these events.

Explicitly modelling prior knowledge lifted *MSH6* to significance however none of the 41 genes with prior probability 0.2 were significant in either frequentist or Bayesian analysis. Bayesian analysis showed significant evidence for 2 additional genes, *VWA1* and *ATAD3C* at a BFDP of 0.0074 however, these both had an adjusted P value of 0.079. Given the convergence of the Bayesian and frequentist analysis, subsequent analysis proceeded with the genes identified in the frequentist analysis; further details are available in Supplementary Tables 5–7.

### Comparison of global CNV burden between endometrial *cancer* cases and controls

The impact of an individual’s CNV burden on endometrial cancer risk was estimated for all CNVs, deletions-only and duplications-only. On average, the total number of CNVs in endometrial cancer cases was 1.22-fold greater than controls (*p* = 4.4 × 10^–63^) and was consistent for CNVs predicted as deletions (fold change [FC] = 1.16, *p* = 1.2 × 10^–25^) and duplications (Table 1, FC = 1.31, *p* = 1.5 × 10^–50^). We further investigated the genomic location of CNVs and estimated the burden of CNVs overlapping genes and exons or in intergenic regions (Table 1). Compared to the burden analysis of total CNVs, the estimated burden was greater for CNVs overlapping genes (FC = 1.30, *p* = 2.1 × 10^–50^) and exons (FC = 1.31, *p* = 7.1 × 10^–48^). In contrast, intergenic CNVs (FC = 1.16, *p* = 1.9 × 10^–32^) displayed reduced burden compared to total CNVs (Table 1).

**Table 1:** Global burden association analysis of rare CNVs

	Mean frequency					
Genomic feature	Cases (n = 4115)	Controls (n = 17,818)	Mean difference	95% CI	p-value^a^	Fold change
**CNVs**						
All	5.99	4.90	1.10	0.97–1.22	4.3 × 10^–63^	1.22
Deletions	3.26	2.80	0.45	0.37–0.54	1.1 × 10^–25^	1.16
Duplications	2.74	2.09	0.64	0.56–0.73	1.4 × 10^–50^	1.31
**Genic CNVs**						
All	2.89	2.22	0.67	0.59–0.76	2.1 × 10^–50^	1.3
Deletions	1.40	1.12	0.28	0.22–0.33	2.2 × 10^–20^	1.25
Duplications	1.49	1.09	0.4	0.34–0.46	2.1 × 10^–38^	1.36
**Exonic CNVs**						
All	2.51	1.92	0.59	0.51–0.67	7.1 × 10^–48^	1.31
Deletions	1.19	0.94	0.25	0.2–0.31	7.0 × 10^–21^	1.27
Duplications	1.32	0.98	0.34	0.28–0.39	4.1 × 10^–34^	1.34
**Intergenic CNVs**						
All	3.10	2.68	0.42	0.35–0.49	1.9 × 10^–32^	1.16
Deletions	1.86	1.68	0.18	0.13–0.23	7.0 × 10^–12^	1.11
Duplications	1.25	1.00	0.25	0.2–0.29	2.6 × 10^–28^	1.25

^a^Student's two-sample *t test*

### Rare CNV association analysis

**Fig. 1:**
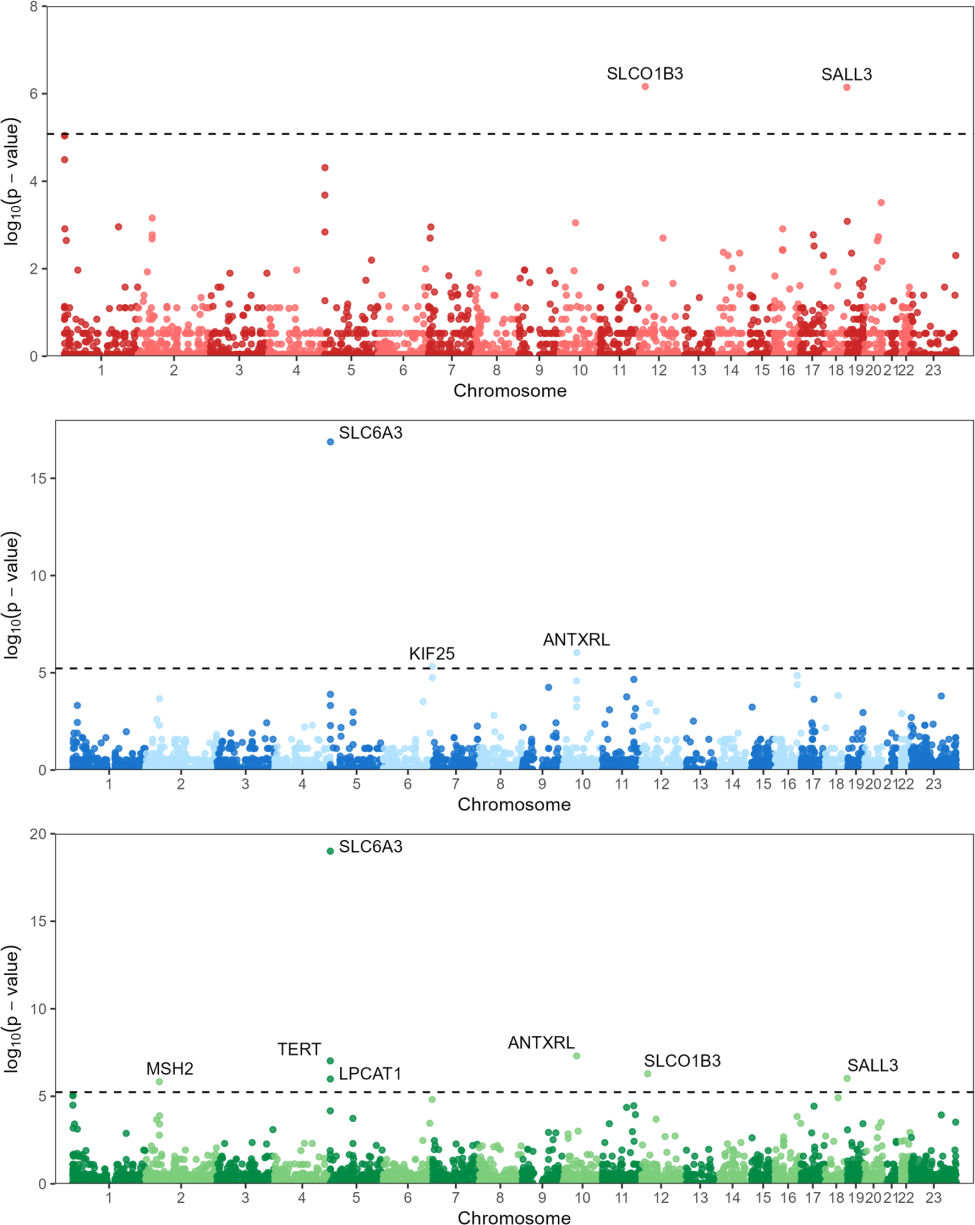
Manhattan plots for CNV-GWAS of 4,115 endometrial cancer cases and 17,818 controls. Genome-wide *p*-values for deletion-only (top), duplication-only (middle) and loss of function CNVs (bottom). Dashed line indicates Bonferroni derived genome-wide significance thresholds at 8.31 × 10^–6^ for deletion-only, 5.97 × 10^–6^ for duplication-only and 5.81 × 10^–6^ for loss of function

To identify specific CNVs associated with endometrial cancer risk, we conducted GWASs for three different association models: a deletion-only, a duplication-only and a loss of function models (all genic deletions and any partial gene duplications) (Supplementary Tables S5-7). We performed gene-centric tests under the assumption that non-overlapping CNVs impacting the same gene locus may have similar effects. The deletion-only model identified a total of 59 gene loci associated (*p* < 0.01) with endometrial cancer, including two loci (*SLCO1B3* and *SALL3*) that met the Bonferroni genome-wide threshold of significance (Fig. 1; Supplementary Table S5). The analysis of duplication variants identified a total of 58 risk-associated loci (*p* < 0.01), including three loci (*SLC6A3, ANTXRL* and *KIF25*) that met genome-wide significance (Fig. 1; Supplementary Table S6). The analysis of loss-of-function variants identified a total of 116 endometrial cancer risk loci (*p* < 0.01), including seven loci (*SLC6A3, ANTXRL, TERT, SLCO1B3, SALL3, LPCAT1* and *MSH2*) that met genome-wide significance (Fig. 1; Supplementary Table S7). Candidate genes (*p* < 0.01) identified by the loss of function model, which includes all deletion variants, captured 93% (55/59) and 64% (37/58) of the candidate genes identified in the deletion-only and duplication-only models, respectively (Supplementary Fig. 1). Additionally, 28 candidate genes were exclusively identified by the loss-of-function model. Only four genes (*LPCAT1, TERT, MSH2* and *SLC6A3*) were identified as candidate risk loci across all three genome-wide association analyses (Supplementary Fig. 1). For each of the genes, all duplications partially overlapped the respective gene boundaries suggesting a shared loss-of-function mechanism with deletions. In total 141 candidate genes (1,525 unique CNVs, *p* < 0.01) were identified across the three association models, including 5 genes (190 unique CNVs) that met genome-wide significance.

### Associations of candidate CNV risk loci at established risk associated SNPs

**Fig. 2:**
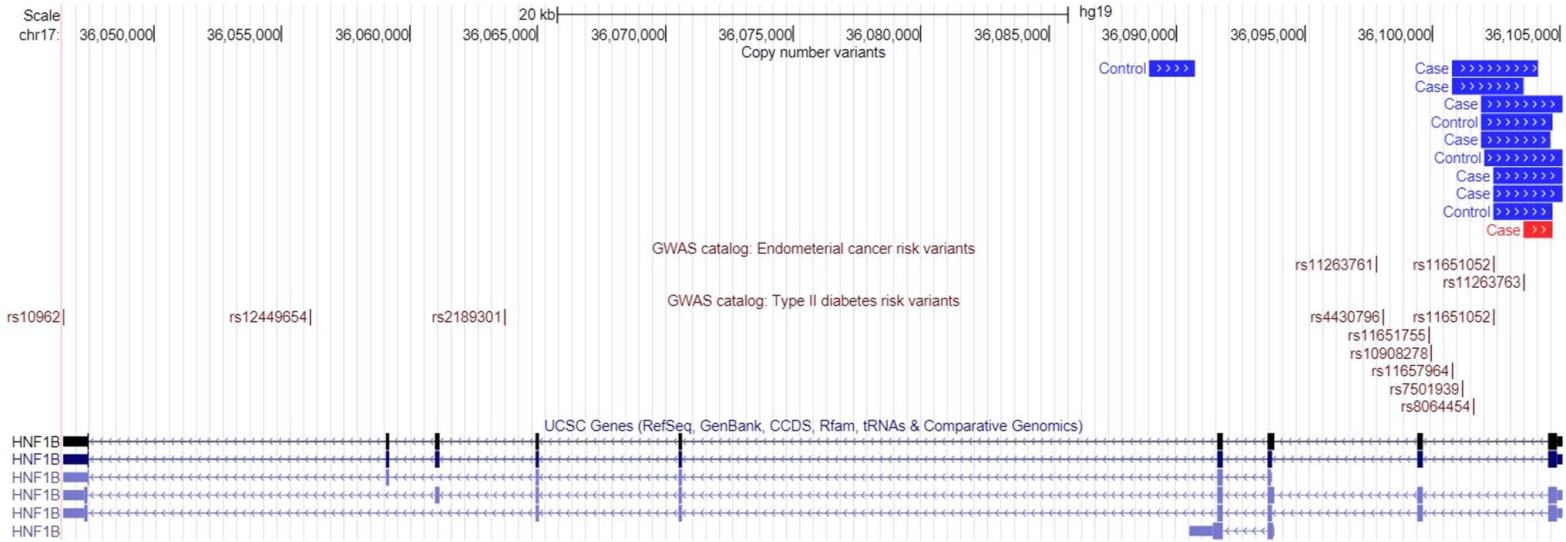
Overlap of putative endometrial cancer risk copy number variants with previously identified endometrial cancer risk and type II diabetes risk variants. Copy number deletions (red) and duplications (blue) in the region of *HNF1B*

We next sought to assess if any of the 1,525 risk-associated candidate CNVs had direct overlap with previously identified GWAS risk SNPs (n = 84) for endometrial cancer risk (Type 2 diabetes [n = 3,516] and obesity [n = 297]). Seven cases and three controls had CNVs that colocalised with two endometrial cancer risk SNPs (rs11263763 and rs11651052) located in intron 1 of *HNF1B* (Fig. 2). Furthermore, CNVs overlapping *HNF1B* were more than six times as frequent in endometrial cancer cases compared to controls (OR = 7.59, 95% CI = 2.29–28.99, *p* = 0.001, Supplementary Table S8). For the traits associated with endometrial cancer risk, 33 Type 2 diabetes-associated and no obesity-associated SNPs were overlapped by at least one candidate endometrial cancer CNV, respectively. Of the 141 candidate gene regions assessed, 50 had at least one CNV overlapping a previously identified risk-SNP. This was driven by a large, multigenic deletion that mapped to the proximal 16p11.2 recurrent breakpoints (BP) 4 and 5 (Supplementary Fig. 2A) that overlaps two Type 2 diabetes risk SNPs (rs8054556 and rs11642340) and 25 risk-associated candidate genes. An additional six lead SNPs had at least one variant in LD (R^2^ > 0.8) that overlapped a risk-associated candidate CNVs. This included three lead SNPs associated with endometrial cancer (rs11263761, rs2278868 and rs882380) and three associated with Type 2 diabetes (rs11651755, rs4430796 and rs8010382). No SNPs associated with obesity (EFO_0001073) from the GWAS Catalog (MacArthur et al. 2017) were found to map to the CNV risk loci.

### Validation of putative rare CNVs

We attempted to validate 17 CNVs (localised to 12 genes), selected from a range of allele frequencies (0.005%-1.49%), in 11 samples using NanoString technology. In total, 12 risk-associated candidate genes were assessed with eight (80%, 8/10) deletions and one (50%, 1/2) duplication validated (Table 2). These data support the reported predictive accuracy of the CamCNV tool (Dennis et al. 2021). This included, validation of three deletions overlapping the known endometrial cancer risk genes (*MSH2* and *PMS2*) in three cases. These three validated CNVs (chr2:47,637,511–47,673,515, chr2:47,639,553–47,639,699, chr7:6,029,431–6029586) overlapped CNVs predicted in a further 26 samples (20 cases, 6 controls). In total, there were 73 CNVs (46 deletions and 27 duplications) overlapping *MLH1, MSH2, MSH6* and *PMS2* in 86 samples (1.28% of cases and 0.19% of controls). A 600 kb deletion at 16p11.2 was validated in one sample (Table 2) using two NanoString probes targeting two different sequences located at chr16:29,653,084–29653175 and chr16:29,875,711–29,875,781. A third probe (16p11_2_389916_32171.1:87) located at chr16:30,125,121–30125192 within the predicted deletion region had insufficient counts (< 100 average counts, Supplementary Table S9). Additionally, two risk-associated deletions overlapping *NPL* (OR_DEL_ = 1.8, *p* = 0.001; Supplementary Table S5) and *SKAP1* (OR_DEL_ = 3.1, *p* = 0.003; Supplementary Table S5) were confirmed in two samples and one sample, respectively.

**Table 2:** Validation results for predicted CNVs

Gene/Loci	MAF^a^	Probes	OR (CI)^b^	*p-value*	Nanostring Validation
**Deletions**					
16p11.2	0.05%	47	6.05 (1.83–23.05)	3.74E–03	100% (1/1)
*CTNNA3*	2.58%	17	1.07 (0.87–1.32)	5.13E–01	100% (1/1)
*MSH2*	0.05%	2–18	6.07 (1.93–19.13)	2.08E–03	100% (2/2)
*MUTYH*	0.02%	7	17.34 (1.93–155.14)	1.07E–02	100% (1/1)
*NPL*	0.65%	3	1.83 (1.27–2.62)	1.10E–03	100% (2/2)
*PMS2*	0.07%	2	4.95 (1.78–13.68)	1.99E–03	100% (1/1)
*FTO*	0.10%	25	2.67 (1.11–6.44)	2.91E–02	100% (1/1)
*SKAP1*	0.13%	13	3.06 (1.46–6.42)	3.02E–03	100% (1/1)
*SALL3*	0.09%	15–19	16.29 (5.40–49.12)	7.16E–07	0% (0/2)
*XRCC1*	0.01%	2–3	8.66 (0.78–95.57)	7.70E–02	0% (0/5)
**Duplications**					
*KIF25*	2.01%	37	1.64 (1.33–2.03)	4.77E–06	100% (2/2)
*SLC6A3*	0.49%	9–15	8.57 (5.23–14.02)	1.34E-17	0% (0/3)

*MAF* minor allele frequency, *OR* odds ratio, *CI* confidence intervals (95%)

^a^Frequencies based on array data

^b^Odds ratios and p-value were calculated using logistic regression

### Pathway analysis of candidate endometrial cancer risk genes

**Fig. 3:**
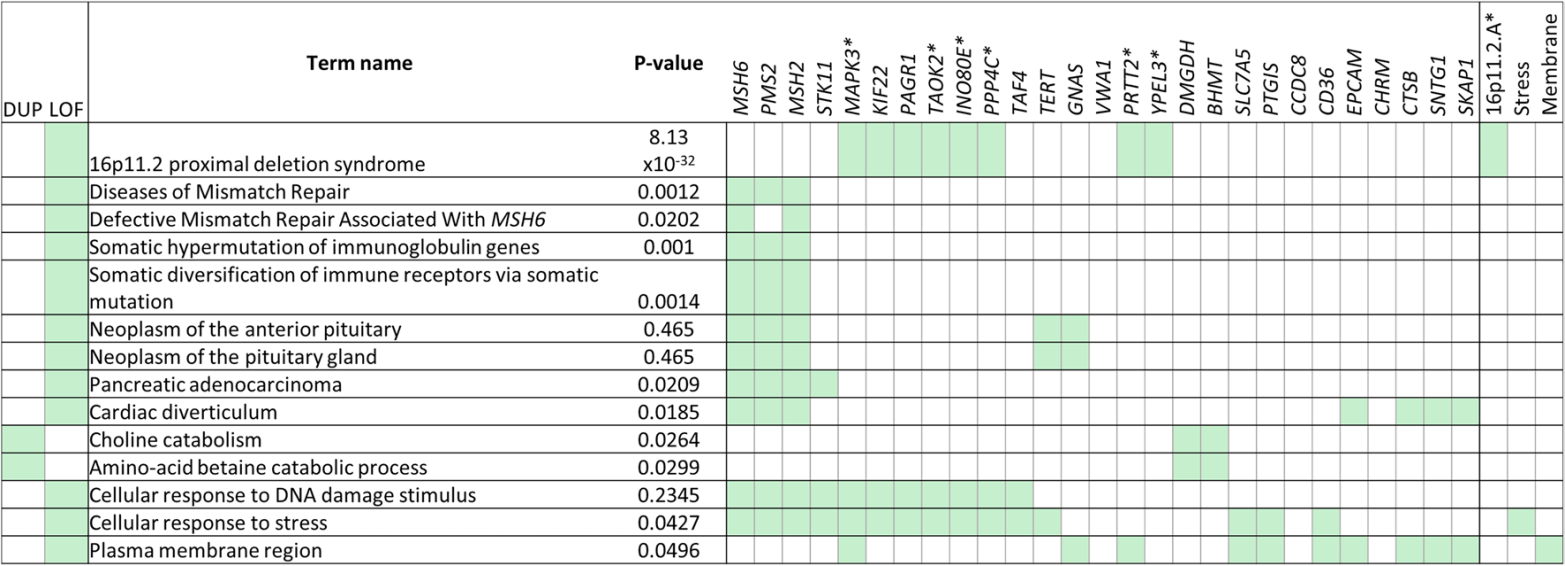
Significantly over-represented pathways for candidate genes derived from duplication-only (DUP) and loss of function (LOF) CNV_GWAS. Significantly enriched pathways are ordered by adjusted p-value (most-to-least significant) of 58 and 116 candidate genes derived from duplication-only and loss of function GWAS. Reactome (REAC) (Fabregat et al., 2018), KEGG (Kanehisa et al., 2019), WikiPathways (WP) (Slenter et al., 2018), Gene Ontology (GO) (Ashburner et al., 2011), Human Phenotype Ontology (HP) (Köhler et al., 2019) were selected as annotation databases. Heatmap on left depicts which CNV-GWAS candidate genes were overrepresented. Gene sets on right side of figure encompass multiple genes: **16p11.2A** = *SPN, QRPT, C16orf54, ZG16, MAZ, MVP, CDIPT, SEZ6L2, ASPHD1, KCTD13, TMEM219, HIRIP3, DOC2A, C16orf92, ALDOA, TBX6, GDPD.* **Stress** = *CYP1B1, FGF12, PPARA, BCLAF1, POLQ, FANCM, ERCC2, GML*. **Membrane** = *SLC6A3, SLCO1B3, DLG2, TMEM231, SLC19A1, SLC4A7.* Genes denoted with * denote additional gene loci identified via recurrent 16p deletion identified in LOF CNV-GWAS but were also represented in other enriched pathways

Due to high degree of overlap between loss of function and deletion-only models (Supplementary Fig. 1), pathway analysis was independently performed on candidate endometrial cancer risk genes for duplication-only and loss-of-function CNV-GWASs (Fig. 3, Supplementary Table S10). The most significantly enriched pathway for loss-of-function CNV-GWAS is 16p11.2 proximal deletion syndrome (MIM: 611,913; *p* = 6.3 × 10^–39^), driven by the recurrent 600 kb long deletion (chr16:29,595,483-30,159,693) identified in six endometrial cancer cases and four controls (0.15% vs 0.02% respectively). This recurrent deletion encompasses 25 genes entirely with 24/25 genes solely impacted by this deletion. The one exception, *MAP3K*, had a single small deletion (28 kb) in one other case sample.

**Fig. 4:**
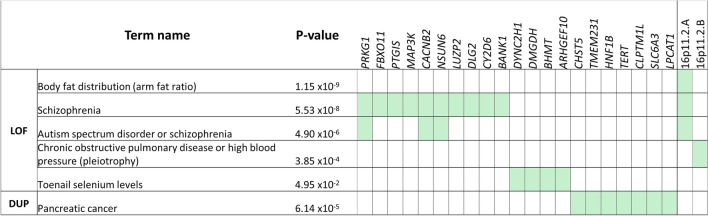
Gene set enrichment analysis for candidate risk genes derived from duplication-only (DUP) and loss of function (LOF) CNV-GWAS. FUMA gene set enrichment analysis results for candidate genes derived from DUP and LOF CNV-GWAS (n = 58 and n = 116, respectively). Adjusted p-values presented. Gene sets on right side encompass two sets of genes, all of which are at 16p11.2 and driven by recurrent deletion identified. **16p11.2.A** = *SEZ6L2, ASPHD1, KCTD13, TMEM219, TAOK2, HIRIP3, INO80E, DOC2A, ALDOA, PPP4C, TBX6, YPEL3, GDPD3*. **16p11.2.B** = *TMEM219, TAOK2, HIRIP3, INO80E, DOC2A, ALDOA, PPP4C*

Moreover, when GWAS-SNP gene sets were tested for enrichment, many of the traits over-represented were driven by those overlapped by this CNV (Fig. 4; Supplementary Table S11). Interestingly, these traits included the enrichment of genes previously linked to body fat distribution (arm fat ratio) (*p* = 1.2 × 10^–9^).

The gene expression data Human Protein Atlas and The Cancer Genome Atlas (TCGA) were used to assess the expression in the endometrium of the genes within the 16p11.2 deletion (n = 25). Additionally, TCGA-UCEC data was used to correlate the expression of each gene with the number of DNA copies (dosage sensitivity, Supplementary Table S12, Supplementary Fig. 3). In normal endometrial tissue, one gene had no detectable expression (*C16orf92*), eight genes had low expression (0.5 < Transcripts per million [TPM] < 10; *ZG16, ASPHD1, TBX6, DOC2A, C16orf54, SPN, KCTD13, GDPD3*) and the remaining 16 exhibited high levels of expression (10 < TPM < 1000). Of the eight ‘low’ expression genes, only the expression levels of *TBX6* and *KCTD13* positively correlated with gene dosage. In contrast, of the more highly expressed genes in normal tissue, all except *TMEM219* and *PRRT2* had a gene dosage effect in endometrial tumour tissue (p < 0.0001). Overall, expression levels correlated positively with gene dosage (*p* < 0.0001) for 16/25 genes in endometrial tumour tissue, supporting the possibility that CNV-related impact on function results in gene expression changes and a potentially abnormal phenotype (Supplementary Table S12; Supplementary Figs. 2b).

## Discussion

While a proportion of endometrial cancer cases that are not currently explained by known genetic risk factors will be explained by epistatic and gene-environment interaction, it is likely that some risk loci have yet to be identified. The objective of this study was to identify rare, germline CNVs that may be associated with endometrial cancer predisposition. We have utilised a large SNP array dataset from the Endometrial Cancer Association and Breast Cancer Association Consortium to conduct a CNV-based GWAS of endometrial cancer. A small proportion (~ 3%) of the cases cohort are likely to be attributed to Lynch Syndrome, however these data were not available within the study cohort (Ryan et al. 2019).

The number of rare CNVs we predicted per individual (5.1 CNVs per individual) is consistent with other case–control studies in breast (5.4 CNVs per individual) and ovarian (5.3 CNVs per individual) cancer cohorts using the same CNV calling methods (Dennis et al. 2022; DeVries et al. 2022). (DE) In our study, endometrial cancer cases had a 1.2-fold greater number of CNVs compared to controls. This is consistent with our previous analyses of a cohort of endometrial cancer cases and controls, that reported an increased burden of rare deletions involving genes and other likely functional regions (Moir-Meyer et al. 2015). However, the increased CNV burden is not observed between ovarian cancer cases and controls (DeVries et al. 2022). The cause of the discrepancy in CNV burden between studies is unclear.

Analysing rare variants is often more challenging than common variants due to the larger sample sizes needed to reach statistical significance (W. Chen et al. 2022). However, the chosen CNV calling method, CamCNV was specifically designed to detect rare variants from genotyping array data while reducing false positives (Dennis et al. 2021). The estimated false discovery rate (FDR) for CNVs called using CamCNV reduces with increasing probe coverage, with the FDR for deletions called by five probes or more estimated at 5.8% and dropping to 1.2% with 10 or more probes. Approximately 53% of deletions were called with ≥ five probes, and 27% of deletions with ≥ 10 probes, increasing our confidence in these findings. Consistent with the estimated FDR for CamCNV, 83% of candidate deletions were validated by Nanostring in deletions called with 2–47 probes. Interestingly, 67% of CNVs called with 2 probes were validated, including two deletions in the Lynch syndrome genes *MSH2* and *PMS2*. CamCNV is less reliable for duplication with a FDR for ≥ 3 probes calls estimated at 62.4% (Dennis et al. 2021). However, approximately 48% of duplications were called using ten or more probes where the FDR is estimated at 8.5%. In this study, we validated three singletons CNVs using NanoString, further increasing our confidence in CamCNVs ability to detect rare, true events.

One way to overcome difficulties associated with rare variant analysis is to perform region-based aggregation tests of multiple variants (Lee et al. 2014). In contrast to SNPs, the impact on a gene by different overlapping CNVs are assumed allowing the aggregation of these CNVs. This approach allowed us to identify genes overlapping with rare CNVs that were associated with endometrial cancer risk, including genes previously implicated in risk by SNP-based association studies. Rare CNVs are over 800 times more likely to be deleterious when compared with single nucleotide variants of the same frequency (Abel et al. 2020). A strength of this study was the loss of function CNV-GWAS in which we tested CNVs based on their likely impact of gene regions. A total of 28 gene regions were found to be significantly associated with endometrial cancer in the loss of function CNV-GWAS. *LPCAT1, TERT, MSH2* and *SLC6A3* were consistently associated with endometrial cancer risk across the three models (deletions-only, duplications-only and loss of function), suggesting a shared loss of function mechanism across CNV type. It is unclear how the loss of function of LPCAT1, TERT or SLC6A3 might contribute to endometrial cancer risk. LPCAT1 is involved in lipid metabolism (Nakanishi et al. 2006) a cellular process which when disrupted may be associated with increased endometrial cancer risk (Rosato et al. 2011). TERT has multiple functions including maintenance of telomere ends, and its activity can have oncogenic effects, such as promoting cell growth and proliferation of cancer cells (Yuan et al. 2019). SLC6A3 functions as a dopamine transporter, as can be found overexpressed in cancers, including renal cell carcinoma and gastric cancer (Hansson et al. 2017).

Our loss of function GWAS recapitulated risk associations identified in endometrial cancer SNP-studies, including variants involving *SKAP1* (O’Mara et al. 2018; Painter et al. 2018). A corresponding transcriptome wide association study (TWAS) demonstrated that decreased expression of *SKAP1* in blood was associated with an increased risk of endometrial cancer (Kho et al. 2021a, b). In this study we report a risk association between loss-of-function variants involving *SKAP1* (OR: 2.4, *p* = 0.008) and endometrial cancer risk, which is consistent with these findings. A novel finding from this study is the association between deletions involving *NPL* and endometrial cancer risk (OR: 1.8, *p* = 0.001). *NPL* regulates intracellular levels of sialic acid, with functional studies demonstrating genetic disruption of *NPL* leads to sialic acid accumulation (Wen et al. 2018). Increased sialic acid levels, or hypersialyation is commonly seen in tumour tissues and leads to accelerated cancer progression (Büll et al. 2014; Dobie & Skropeta 2021; Sun et al. 2020). Moreover, high levels of sialyation in endometrial cells has been shown to promote endometriosis outbreaks via TGF- β1 (Choi et al. 2018). Given the shared biological aetiology between endometrial cancer and non-cancerous gynaecological diseases such as endometriosis (Kho et al. 2021a, b; Painter et al. 2018), the association identified between deletions involving *NPL* and endometrial cancer risk warrants further investigation.

Obesity traits are well established risk factors for endometrial cancer (Aune et al. 2015; Painter et al. 2016), at least partly due to the accumlation of unopposed oestrogen (Lukanova et al. 2004). In this study, pathway enrichment analyses of candidate endometrial cancer risk genes revealed a strong over-representation of genes involved in 16p11.2 proximal deletion syndrome (MIM: 611,913), that is characterised by clinical heterogeneity and incomplete penetrance (Fetit et al. 2020). Proximal 16p11.2 BP4-BP5 deletions are highly pleiotropic and have been associated with many neurocognitive phenotypes, neurological tumours, morbid obesity and epilepsy (Auwerx et al. 2024; Bijlsma et al. 2009; Egolf et al. 2019; Fetit et al. 2020; Jacquemont et al. 2011; Shinawi et al. 2010; Ventura et al. 2019). This is consistent with genetic correlation between obesity traits and endometrial cancer risk (O’Mara et al. 2018). Repetitive regions at 16p11.2 result in recurrent structural changes, the most common of which being a proximal 16p11.2 BP4-BP5 deletion at chr16: 29.6—30.2 Mb (Zufferey et al. 2012). We observed a risk-associated deletion among ten women at this locus, that is completely retained within this clinically defined region. Microdeletions at 16p11.2 result in a predisposition to obesity, with reciprocal deletions and duplications being respectively associated with obesity and being underweight, highlighting a gene dosage mechanism (Bochukova et al. 2010; Jacquemont et al. 2011; Macé et al. 2017; Walters et al. 2010). Expression levels for some but not all genes within the proximal 16p11.2 BP4-BP5 have previously been shown to correlate with copy number in pluripotent stem cells, lymphoblastoid cell lines and adipose tissues (Jacquemont et al. 2011; Roth et al. 2020; Walters et al. 2010). To our knowledge, this is the first time the relationship between gene copy and expression of genes involved in this deletion have been assessed in endometrial tissue and our results suggest potential dosage effects for the majority of genes assessed. Interestingly, the transcription factor *TBX6* is expressed at low levels in normal endometrial tissue but a correlation between *TBX6* gene dosage and expression was identified in endometrial tumour tissue. *TBX6* has been implicated as a candidate gene for another associated clinical manifestation of microdeletions at 16p11.2 which leads to a complete absence, or underdevelopment, of the female reproductive system (with Mayer-Rokitansky-Küster-Hauser syndrome [MRKH; MIM: 277000]). Studies have reported a significant association of 16p11.2 deletions among individuals with MRKH, potentially indicating that genes near this locus are involved in uterine development (Chen et al. 2021; Gatti et al. 2018). Results from this study support loss-of-function at this region is associated with endometrial cancer risk, with possible risk mechanisms being linked to obesity and/or uterine development.

Despite this being the largest endometrial cancer CNV-dataset analysed to date, the rarity of the CNVs identified results in limited power for detecting significant associations. We therefore used a nominal threshold of *p* < 0.01 to prioritise gene regions as candidate risk genes. Explicitly modelling prior associations with a generous prior did not materially alter our results providing some assurance that the genome wide adjustment used in our standard analysis is best practise, at least with our current knowledge of the genomic landscape of endometrial cancer. With this current study we aimed to identify a broad array of candidates, and thus all results reported on require further validation in independent datasets. We acknowledge that this is a limitation of the study, however in silico assessment and prioritisation was employed as a way to compliment the empirical approach. Pathway analysis of candidate genes revealed an enrichment of obesity and cancer pathways and identified multiple genes/loci that warrant further investigation.

In summary, we have conducted the largest CNV-GWAS for endometrial cancer predisposition. We have shown a global burden of rare CNVs and support the association between increased genomic load of rare CNVs and endometrial cancer risk. Our prioritisation workflow led to the identification of 141 candidate endometrial cancer susceptibility genes, many of which have plausible biological mechanisms to suggest an involvement in endometrial cancer susceptibility. Clinical features previously associated with proximal 16p11.2 BP4-BP5 deletions, including predisposition to obesity and congenital reproductive tract development, make this a particularly intriguing risk association that warrants further study.

## Supplementary Information

Below is the link to the electronic supplementary material.

Supplementary file1 (XLSX 6898 KB)

Supplementary file2 (PDF 617 KB)

## Data Availability

OncoArray germline genotype data for the ECAC studies have been deposited through the database of Genotypes and Phenotypes (dbGaP; accession number phs000893.v1.p1). Genotype data for non-cancer controls were provided by the Breast Cancer Association Consortium (BCAC) by application to the BCAC Data Access Coordination Committee (http://bcac.ccge.medschl.cam.ac.uk/).
