# Supplementary material for: Germline copy number variants and endometrial cancer risk
Source: Hum Genet. 2024 Nov 4;143(12):1481–98. doi: 10.1007/s00439-024-02707-9 (PMC11576655; doi:10.1007/s00439-024-02707-9)
Supplement: Supplementary file 2 — Supplementary file2 (PDF 617 KB) [file 439_2024_2707_MOESM2_ESM.pdf]

Germline copy number variants and endometrial cancer risk

Cassie E. Stylianou[1*], George A.R. Wiggins[1*], Vanessa L. Lau[1], Joe Dennis[2], Andrew N. Shelling[3], Michelle Wilson[4], Peter Sykes[5], Frederic Amant[6, 7], Daniela Annibali[7], Wout De Wispelaere[7], Douglas F. Easton[2, 8], Peter A. Fasching[9], Dylan M. Glubb[10], Ellen L. Goode[11], Diether Lambrechts[12, 13], Paul D.P. Pharoah[14], Rodney J. Scott[15-17], Emma Tham[18, 19], Ian Tomlinson[20], Manjeet K. Bolla[2], Fergus J. Couch[21], Kamila Czene[22], Thilo Dörk[23], Alison M. Dunning[8], Olivia Fletcher[24], Montserrat García-Closas[25], Reiner Hoppe[26, 27], ABCTB Investigators[28], Helena Jernström[29], Rudolf Kaaks[30], Kyriaki Michailidou[2, 31], Nadia Obi[32, 33], Melissa C. Southey[34-36], Jennifer Stone[37, 38], Qin Wang[2], Amanda B. Spurdle[39], Tracy A. O'Mara[10], John Pearson[40], Logan C. Walker[1]

* These authors contributed equally to this work

[1] Department of Pathology and Biomedical Science, University of Otago, Christchurch, New Zealand.
[2] Centre for Cancer Genetic Epidemiology, Department of Public Health and Primary Care, University of Cambridge, Cambridge, UK.
[3] Department of Obstetrics and Gynaecology, University of Auckland, Auckland, New Zealand.
[4] Te Pūriri o Te Ora Regional Cancer and Blood Service, Auckland Hospital, Auckland, New Zealand.
[5] Department of Obstetrics and Gynaecology, University of Otago, Christchurch, New Zealand.
[6] Department of Obstetrics and Gynecology, Division of Gynecologic Oncology, University Hospitals KU Leuven, University of Leuven, Leuven, Belgium.
[7] Gynecological Oncology Laboratory, Department of Oncology, KU Leuven and Leuven Cancer Institute (LKI), Leuven, Belgium.
[8] Centre for Cancer Genetic Epidemiology, Department of Oncology, University of Cambridge, Cambridge, UK.
[9] Department of Gynecology and Obstetrics, Comprehensive Cancer Center Erlangen-EMN, Friedrich-Alexander University Erlangen-Nuremberg, University Hospital Erlangen, Erlangen, Germany.
[10] Cancer Research Program, QIMR Berghofer Medical Research Institute, Brisbane, Queensland, Australia.
[11] Department of Quantitative Health Sciences, Division of Epidemiology, Mayo Clinic, Rochester, MN, USA.
[12] Laboratory for Translational Genetics, Department of Human Genetics, KU Leuven, Leuven, Belgium.
[13] VIB Center for Cancer Biology, VIB, Leuven, Belgium.
[14] Department of Computational Biomedicine, Cedars-Sinai Medical Center, West Hollywood, CA, USA.
[15] Division of Molecular Medicine, Pathology North, John Hunter Hospital, Newcastle, New South Wales, Australia.
[16] Discipline of Medical Genetics, School of Biomedical Sciences and Pharmacy, Faculty of Health, University of Newcastle, Callaghan, New South Wales, Australia.
[17] Hunter Medical Research Institute, John Hunter Hospital, Newcastle, New South Wales, Australia.
[18] Department of Molecular Medicine and Surgery, Karolinska Institutet, Stockholm, Sweden.
[19] Clinical Genetics and Genomics, Karolinska University Hospital, Stockholm, Sweden.
[20] Department of Oncology, University of Oxford, Oxford, UK.
[21] Department of Laboratory Medicine and Pathology, Mayo Clinic, Rochester, MN, USA.
[22] Department of Medical Epidemiology and Biostatistics, Karolinska Institutet, Stockholm, Sweden.
[23] Gynaecology Research Unit, Hannover Medical School, Hannover, Germany.
[24] The Breast Cancer Now Toby Robins Research Centre, The Institute of Cancer Research, London, UK.
[25] Division of Genetics and Epidemiology, The Institute of Cancer Research, London, UK.
[26] Dr. Margarete Fischer-Bosch-Institute of Clinical Pharmacology, Stuttgart, Germany.
[27] University of Tübingen, Tübingen, Germany.

[28] Australian Breast Cancer Tissue Bank, Westmead Institute for Medical Research, University of Sydney, Sydney, New South Wales, Australia.
[29] Oncology, Department of Clinical Sciences in Lund, Lund University, Lund, Sweden.
[30] Division of Cancer Epidemiology, German Cancer Research Center (DKFZ), Heidelberg, Germany.
[31] Biostatistics Unit, The Cyprus Institute of Neurology and Genetics, Nicosia, Cyprus.
[32] Institute for Occupational and Maritime Medicine, University Medical Center Hamburg-Eppendorf, Hamburg, Germany.
[33] Institute for Medical Biometry and Epidemiology, University Medical Center Hamburg-Eppendorf, Hamburg, Germany.
[34] Precision Medicine, School of Clinical Sciences at Monash Health, Monash University, Clayton, Victoria, Australia.
[35] Department of Clinical Pathology, The University of Melbourne, Melbourne, Victoria, Australia.
[36] Cancer Epidemiology Division, Cancer Council Victoria, Melbourne, Victoria, Australia.
[37] Genetic Epidemiology Group, School of Population and Global Health, University of Western Australia, Perth, Western Australia, Australia.
[38] Centre for Epidemiology and Biostatistics, Melbourne School of Population and Global Health, The University of Melbourne, Melbourne, Victoria, Australia.
[39] Public Health Program, QIMR Berghofer Medical Research Institute, Brisbane, Queensland, Australia.
[40] Department of Medicine, University of Otago, Christchurch, New Zealand.

Corresponding Author: George Wiggins

Address: Department of Pathology and Biomedical Science, University of Otago Christchurch, New Zealand

Telephone: (643) 364 0557

Email: george.wiggins@otago.ac.nz

CNV-GWAS	Genes $p < 0.01$
Deletion-only	59
Duplication-only	58
Loss of function	116

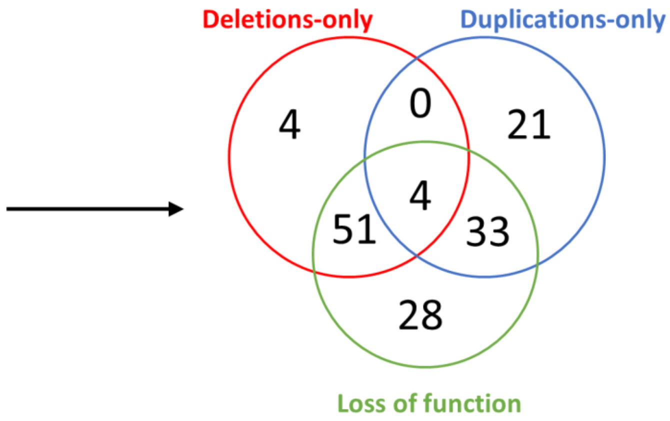

Supplementary Figure 1.

Table shows number of genes with p <0.01 from each of the three CNV-GWAS. Venn diagram shows the concordance between candidate genes.

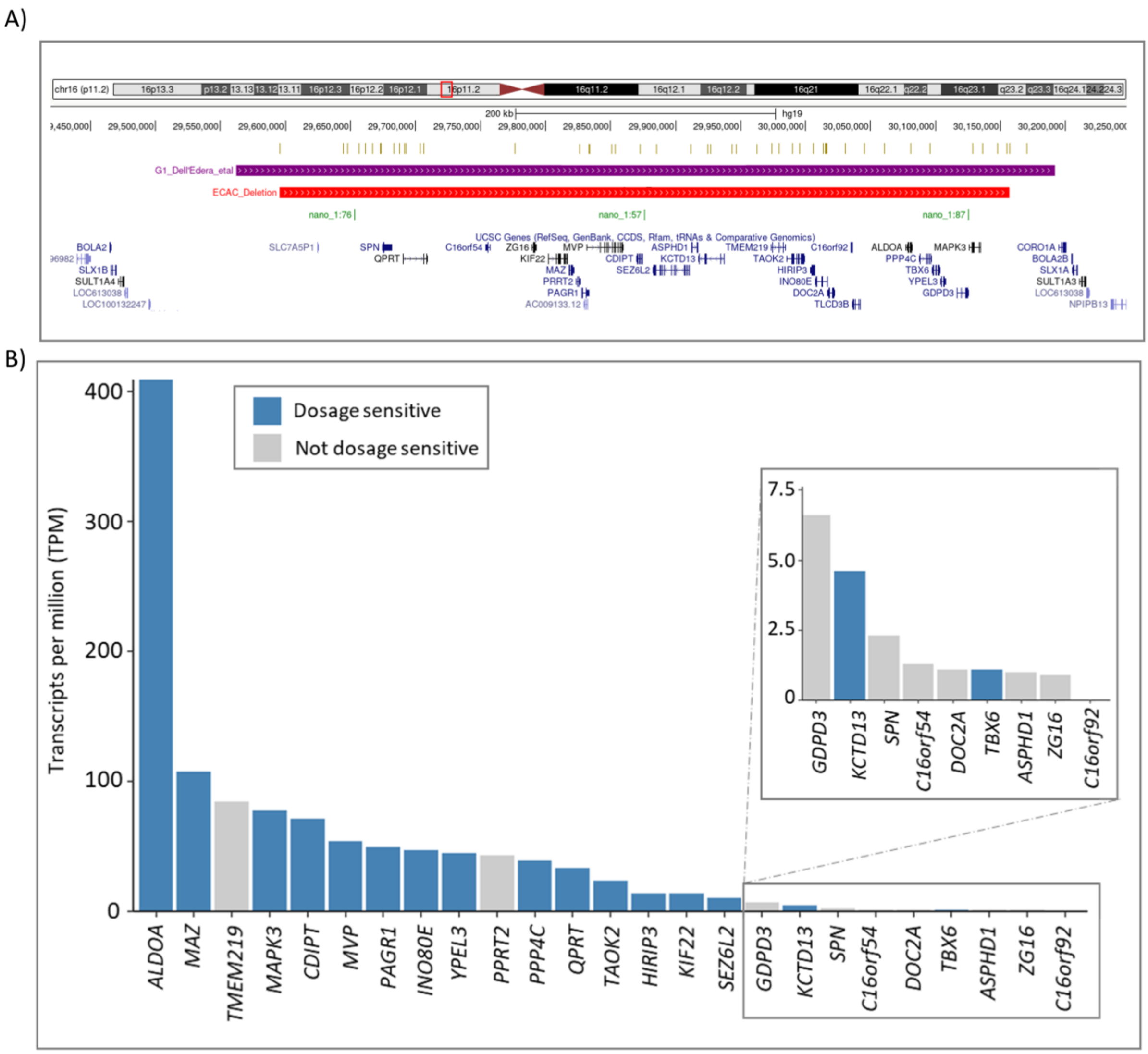

Supplementary Figure 2.

A) Germline deletions represented by red bars, duplications by blue bars. NanoString probe location presented in dark green (track name: NanoString) Genes encompassed by the ECAC CNV (red) and published syndromic CNV (purple) are shown. Genomic positions according to hg19 genomic build. B) Expression in normal endometrium tissue. Coloured bars represent those genes that show evidence ($p < 0.0001$) of dosage sensitivity in endometrial cancer tissue.

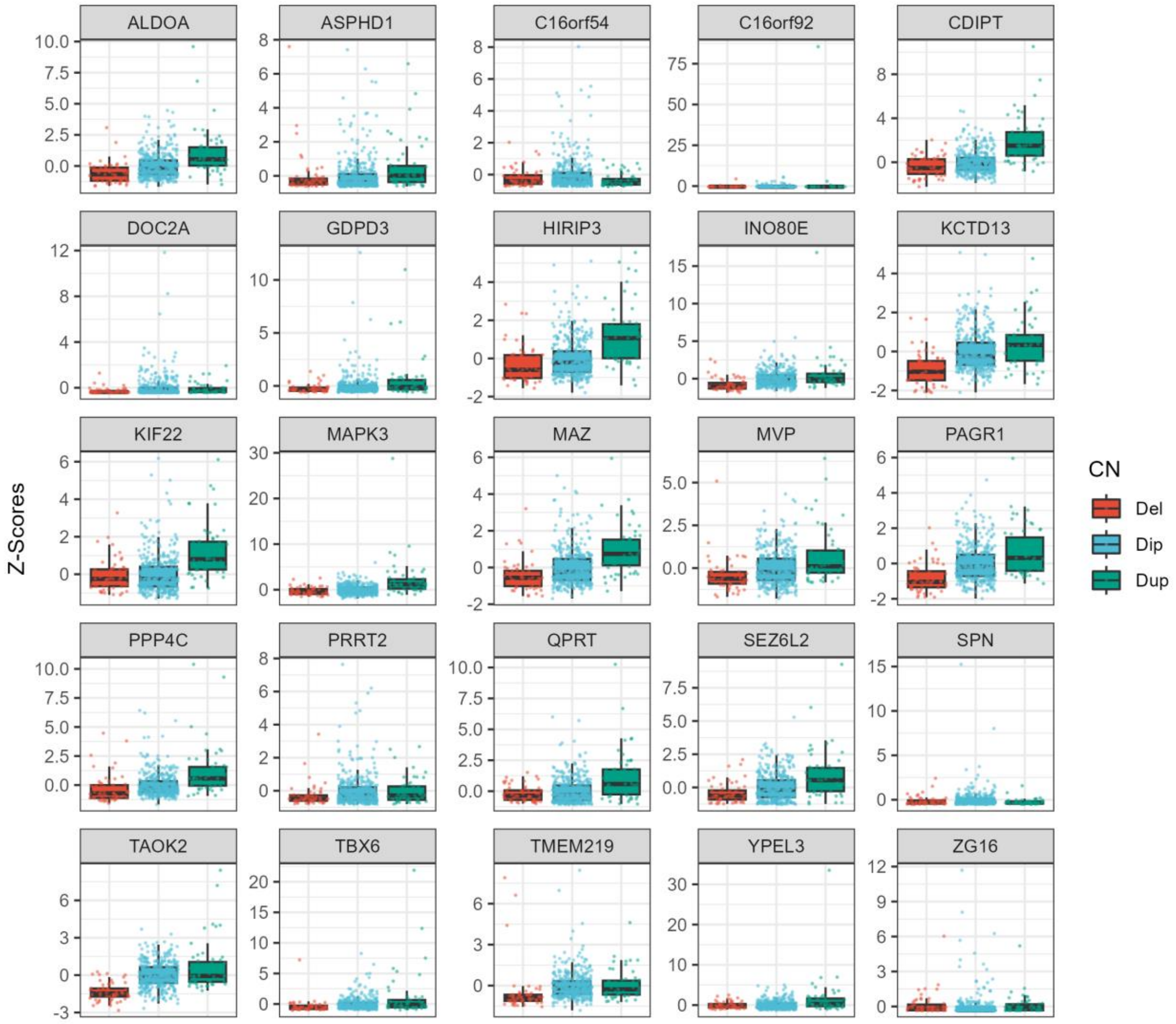

Supplementary Figure 3

Gene expression and dosage of 24 genes at the proximal 16p11.2 BP4-BP5 locus in 521 endometrial tumours from TCGA. Sample are grouped based on copy number status. Del, deletion (red); Dip, diploid (blue); Dup, duplication (green).
